# Comprehensive assessments of pulmonary circulation in children with pulmonary hypertension associated with congenital heart disease

**DOI:** 10.3389/fped.2022.1011631

**Published:** 2022-10-14

**Authors:** Jun Muneuchi, Hiroki Ezaki, Yuichiro Sugitani, Mamie Watanabe

**Affiliations:** Department of Pediatrics, Kyushu Hospital, Japan Community Healthcare Organization

**Keywords:** pulmonary arterial hypertension, pulmonary vascular resistance, pulmonary arterial capacitance, pulmonary arterial compliance, time constant (tau), resistor–capacitor time

## Abstract

Pulmonary hypertension associated with congenital heart disease (CHD-PH) encompasses different conditions confounded by the left-to-right shunt, left heart obstruction, ventricular dysfunction, hypoxia due to airway obstruction, dysplasia/hypoplasia of the pulmonary vasculature, pulmonary vascular obstructive disease, and genetic variations of vasoactive mediators. Pulmonary input impedance consists of the pulmonary vascular resistance (Rp) and capacitance (Cp). Rp is calculated as the transpulmonary pressure divided by the pulmonary cardiac output, whereas Cp is calculated as the pulmonary stroke volume divided by the pulmonary arterial pulse pressure. The plots of Rp and Cp demonstrate a unique hyperbolic relationship, namely, the resistor–capacitor coupling curve, which represents the pulmonary vascular condition. The product of Rp and Cp is the exponential pressure decay, which refers to the time constant. Alterations in Cp are more considerable in CHD patients at an early stage of developing pulmonary hypertension or with excessive pulmonary blood flow due to a left-to-right shunt. The importance of Cp has gained attention because recent reports have shown that low Cp potentially reflects poor prognosis in patients with CHD-PH and idiopathic pulmonary hypertension. It is also known that Cp levels decrease in specific populations, such as preterm infants and trisomy 21. Therefore, both Rp and Cp should be individually evaluated in the management of children with CHD-PH who have different disease conditions.

## Introduction

In contrast to the systemic circulation, the pulmonary circulation is characterized by a low-pressure system despite receiving cardiac output similar to that of the systemic circulation. Whenever cardiac output during exercise increases by 5–6 times more than at rest, pulmonary arterial pressure remains stable with subtle elevation during exercise (1, 2), suggesting that pulmonary circulation exhibits extreme high compliance. Therefore, pulmonary hypertension indicates an excessive increase in pulmonary blood flow or a significant reduction in the effective pulmonary vascular bed. Regarding causes of pulmonary hypertension, the World Symposium on Pulmonary Hypertension has offered five clinical classifications, or the Nice classification, which consists of the following: (1) pulmonary arterial hypertension, (2) pulmonary hypertension due to left heart disease, (3) pulmonary hypertension due to lung disease and/or hypoxemia, (4) chronic thromboembolic pulmonary hypertension, and (5) pulmonary hypertension with unclear or multifactorial mechanisms (3). In terms of pulmonary hypertension associated with congenital heart disease (CHD-PH), the European Society of Cardiology provides four main clinical subgroups: (1) Eisenmenger syndrome, (2) pulmonary arterial hypertension associated with systemic-to-pulmonary shunts, (3) pulmonary arterial hypertension with small defects, and (4) pulmonary arterial hypertension after corrective cardiac surgery (4). These clinical classifications are helpful in understanding the pathophysiology of CHD-PH. However, CHD-PH encompasses different conditions confounded by the left-to-right shunt, left heart obstruction, ventricular dysfunction, hypoxia due to airway obstruction, dysplasia/hypoplasia of the pulmonary vasculature, pulmonary vascular obstructive disease, and genetic variations of vasoactive mediators and growth factors. Therefore, multidisciplinary approaches are necessary to assess pulmonary circulation in patients with CHD-PH.

Despite advances in imaging modalities such as echocardiography, computed tomography, and magnetic resonance imaging, invasive right heart catheterization remains a mainstay for evaluating pulmonary circulation because it allows direct measurement of pulmonary arterial pressure, pulmonary arterial wedge pressure, and pulmonary blood flow amount. Pulmonary vascular resistance (Rp), calculated based on pulmonary arterial pressure and pulmonary blood flow, has been established as a standard pulmonary hemodynamic parameter in routine right heart catheterization. Most pediatric cardiologists and cardiovascular surgeons still believe that these parameters are sufficient to evaluate pulmonary circulation in patients with CHD-PH. However, pulmonary circulation works as a series of resister–capacitor circuits and consists of resistant and capacitance vessels. Although Rp only reflects the nonpulsatile component of pulmonary blood flow, the importance of pulmonary arterial capacitance (Cp), which reflects the pulsatile component, tends to be overlooked. Therefore, both Rp and Cp must be considered when managing patients with CHD-PH. Thus, we describe a comprehensive assessment of pulmonary circulation, including Rp and Cp, in patients with CHD-PH.

## Components of the pulmonary arterial input impedance

**Figure 1:**
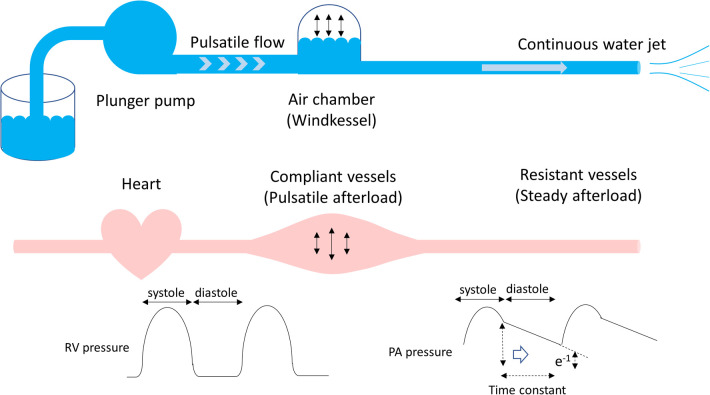
Windkessel model. The rhythmic water output of the plunger strokes is transformed into a continuous water jet downstream through an air tank. In terms of the pulmonary circulation, the plunger pump, an air tank, and a duct represent the heart, compliant vessels, and resistant vessels, respectively. The pulmonary arterial input impedance consists of compliant and resistant vessels, which determine the right ventricular afterload. The product of Rp and Cp is time-constant, which is an exponential pressure decay in diastole, referring time until pulmonary arterial pressure decreases to e^−1^ (37%) of pulmonary arterial end-systolic pressure. Rp, pulmonary vascular resistance; Cp, pulmonary vascular capacitance.

The right ventricle ejects blood against the pulmonary arterial load, namely, the pulmonary arterial input impedance, which can be divided into steady (hydraulic) and pulsatile components. The best-simplified description of the pulmonary arterial load is the Windkessel model proposed by Dr. Otto Frank in 1899 (5), wherein the rhythmic water output of the plunger strokes is transformed into a continuous water jet downstream through an air tank (Windkessel). This model comprises physiologically interpretable parameters in terms of vascular resistance and compliance (Figure 1). Resistance refers to the viscous and inertial properties of the vascular bed, whereas compliance or capacitance refers to the elastic properties of the entire arterial system. As pulmonary circulation is a low-pressure and highly compliant system, both Rp and Cp are important determinants of the pulmonary arterial input impedance.

In patients with CHD, anatomical defects, such as ventricular septal defect or patent ductus arteriosus, influence pulmonary blood flow volume in accordance with alterations in pulmonary arterial input impedance. Pulmonary arterial input impedance is physiologically altered by a postnatal reduction in Rp and alveolar hypoxic vasoconstriction due to airway problems. Additionally, a persistent increase in pulmonary blood flow leads to an increase in vascular shear stress and progressive endothelial dysfunction, resulting in the remodeling of the pulmonary vasculature, such as medial thickness or intimal hyperplasia. Although early corrective surgery can avoid the development of these histopathological changes in the pulmonary vasculature, delayed surgery leads to intractable and irreversible histopathological changes, resulting in a reversal shunt through a defect, which is referred to as Eisenmenger syndrome.

## Pulmonary vascular resistance and capacitance

Rp is commonly determined by routine right heart catheterization in patients with CHD and is calculated as follows:

$$\mathrm{Rp}=\frac{\left(\mathrm{mean}\;\mathrm{PAP}\text{–}\mathrm{PCWP}\right)}{\mathrm{Qp}}$$

where PAP and PCWP represent the pulmonary arterial pressure and pulmonary capillary wedge pressure, respectively. In CHD patients with an interatrial defect, such as patent foramen ovale or atrial septal defect, PCWP can be replaced by mean left atrial pressure or pulmonary venous pressure. Measurement of pulmonary venous pressure is reliable and preferable in patients with CHD-PH because they occasionally accompany pulmonary venous obstruction. During right heart catheterization in patients with CHD, it should always be considered whether the measurement condition and sampling sites are appropriate since the pulmonary blood flow amount, which is conventionally determined based on the Fick principle, directly affects the results of Rp.

Cp is another important parameter that determines pulmonary circulation. Cp is calculated by the following formula:

$$\mathrm{Cp}=\frac{\mathrm{SV}}{\mathrm{PP}}$$

where SV represents the stroke volume and PP represents pulmonary pulse pressure, which is calculated as pulmonary systolic pressure minus pulmonary diastolic pressure. The other method to calculate Cp is derived from the pressure decay directly measured based on the waveform of pulmonary arterial pressure, which is likely to be cumbersome because it requires analysis equipment. Pulmonary arterial compliance and capacitance (Cp) have been discussed synonymously in previously published literature works. Although capacitance is calculated as the above formula, compliance is calculated as an absolute change in the pulmonary arterial lumen area divided by PP. Calculating compliance has a disadvantage because the measurement of cross-sectional areas in the pulmonary arteries is necessary. Sanz et al. described several parameters for evaluating the stiffness of the pulmonary arteries, including pulsatility, compliance, distensibility, elastic modulus, stiffness index *β*, and Cp (Table 1) (6). Among them, Cp is inversely proportional to the energy consumption of the right ventricle and is the most convenient parameter during conventional right heart catheterization because it is calculated simply based on pulmonary arterial pressure and pulmonary blood flow amount (7). In general, it is difficult to calculate the precise value of Cp because blood leaves the arterial system through the peripheral vessels during cardiac ejection (8). However, in practice, the pulse pressure method is acceptable for calculating Cp in accordance with reliable data (9–11).

**Table 1:** Indices of pulmonary arterial stiffness.

Parameters	Units	Formula	Definition
Pulsatility	%	[(maxA − minA)/minA] × 100	Relative change in lumen area during the cardiac cycle
Compliance	mm^2^/mmHg	(maxA − minA)/PP	Absolute change in lumen area for a given change in pressure
Capacitance	ml/mmHg	SV/(sPAP − dPAP)	Change in volume associated with a given change in pressure
Distensibility	%/mmHg	[(maxA − minA)/PP × minA] × 100	Relative change driving a relative increase in lumen area
Elastic modulus	mmHg	PP × minA/(maxA − minA)	Pressure change driving a relative increase in lumen area
Stiffness index *β*	–	ln(sPAP/dPAP)/[(maxA − minA)/minA]	Slope of the function between distending arterial pressure and arterial distension

dPAP, diastolic pulmonary arterial pressure; maxA, maximum area of the pulmonary artery; minA, minimum area of the pulmonary artery; sPAP, systolic pulmonary arterial pressure; SV, stroke volume. Revised according to reference (6).

A normal Cp value in healthy adult individuals is reported to be 5.5 ± 1.6 ml/mmHg (approximately 3.2 ± 0.9 ml/mmHg/m^2^) (12). Although the normal value of Cp in children is unknown, Cp is reported to be 2.7 ml/mmHg/m^2^ (ranging from 2.2 to 3.3 ml/mmHg/m^2^) in infants with ventricular septal defect and pulmonary hypertension, which seems to be slightly lower than the normal Cp value in adults (13). In addition, Cp can be altered under different disease conditions. Sajan et al. reported that Cp was significantly lower in children with idiopathic pulmonary hypertension than those in children with CHD-PH (1.09 ± 0.68 vs. 1.87 ± 1.62, *P* < 0.045) (14).

**Figure 2:**
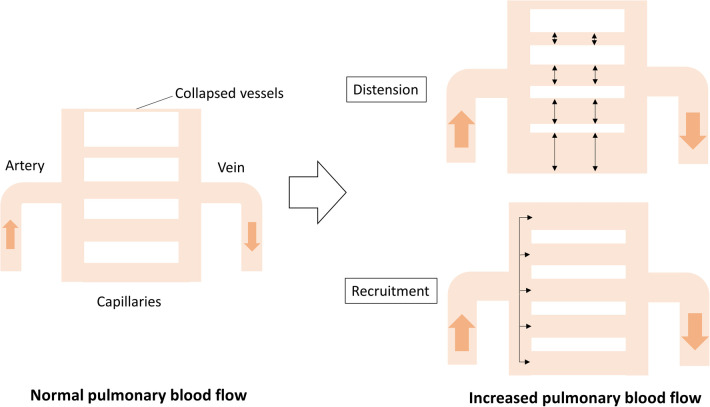
Cp determined by distention and recruitment of pulmonary vessels including capillaries. When cardiac output increases, the blood flow is accommodated by distention and recruitment, ensuring that the pulmonary circulation is highly compliant. Cp, pulmonary vascular capacitance.

It is postulated that Cp consists of the distensibility and recruitment of pulmonary vessels, including capillaries (Figure 2). The distensibility of the vessels represents stiffness; however, the stiffness of the proximal pulmonary arteries, including the main pulmonary trunk and hilar branches, contributes to only 10%–15% of the total Cp (15). Therefore, the distensibility and recruitment of intrapulmonary capillaries are more likely to contribute to Cp. Recruitment of pulmonary capillaries is another determinant of Cp. Pulmonary capillaries partially or completely collapse in the setting of normal pulmonary blood flow, whereas these collapsed vessels are recanalized according to an increase in pulmonary blood flow due to left-to-right shunt. This recruitment attributes to the fact that pulmonary capillaries are exceedingly thin and compliant (16). Langelben et al. reported that an increase in pulmonary blood flow is mainly accommodated by recruitment, with progressive distention according to a further increase in animal models (17). In patients with CHD-PH, Cp rather than Rp can predict the development of pulmonary vascular diseases, such as medial thickness or hypoplastic arterioles, suggesting that a low Cp reflects an early stage of reduced effective pulmonary vascular beds following impaired recruitment and distensibility of the pulmonary capillaries (18).

Recently, Cp has gained attention in the management of patients with different conditions of pulmonary arterial hypertension (Table 2) (13, 14, 18–29). Douwes et al. reported that a higher Cp was associated with improved survival, independent of the World Health Organization-functional class and therapy in children with CHD-PH and idiopathic pulmonary arterial hypertension (20). Takastuki et al. reported that Cp was inversely correlated with brain natriuretic peptide levels and the New York Heart Association functional class in children with idiopathic or hereditary pulmonary arterial hypertension, which served as a poor prognostic marker (22). These findings are consistent with previous reports on adult pulmonary arterial hypertension (30, 31). Cheng et al. reported that Cp was an independent predictor of poor prognosis among heart rate, Rp, Cp, and the 6-min walk test in 111 adult patients with CHD-PH, including 48 patients with Eisenmenger syndrome (24). Iwaya et al. reported that low Cp could predict poor prognosis in infants with isolated atrial septal defects and severe pulmonary hypertension who exhibited fatal courses (26). These findings suggest that low Cp rather than high Rp potentially reflects a poor prognosis in children with CHD-PH.

**Table 2:** List of the literature works regarding pulmonary vascular resistance and capacitance in patients with CHD-PH or children with idiopathic PAH.

Authors/published year	Category	Subjects’ number and age	Results of parameters	Summary
Basnet, 2000 (19)	ASD/VSD	*N* = 124 45 days–12 years	mPAP: 16 (9–69) mmHg Rp: 0.12 ± 0.01 mmHg/ml m^2^ Cp: 1.53 ± 0.17 ml/mmHg m^2^	Children with VSD/PH exhibited lower Cp than children with VSD or ASD/no PH. Cp was higher in female VSD patients than that in male VSD patients.
Sajan, 2010 (14)	Idiopathic PAH	*N* = 19 7.1 ± 6.2 years	mPAP: 41 ± 18 mmHg Rp: 12.34 ± 7.21 Wood units m^2^ Cp: 1.87 ± 1.62 ml/mmHg m^2^	Low Cp and high Rp were associated with 6 MWD and a freedom from death or transplant. A Cp <0.70 ml/mmHg m^2^ or >1.25 ml/mmHg m^2^ and an Rp >13 Wood units m^2^ were associated with decreased freedom from death or lung transplant
	CHD-PAH	*N* = 28 8.4 ± 5.5 years	mPAP: 57 ± 25 mmHg Rp: 20.75 ± 12.58 Wood units m^2^ Cp: 1.09 ± 0.68 ml/mmHg m^2^	
Douwes, 2013 (20)	Idiopathic PAH	*N* = 32 7.6 (3.8–14.0) years	mPAP: 51 ± 20 mmHg Rp: 14.0 (7.1–26.9) Wood units m^2^ Cp: 0.9 (0.6–1.2) ml/mmHg m^2^	PAH patients, irrespective idiopathic PAH or CHD-PAH, had significantly lower Cp compared to control subjects. Low Cp was associated with poor WHO-functional class, whereas high Cp was associated with improved survival, independent from WHO-functional class and PAH-targeted therapy
	CHD-PAH	*N* = 20 7.0 (2.0–11.8) years	mPAP: 51 ± 16 mmHg Rp: 14.6 (8.3–19.0) Wood units m^2^ Cp: 0.9 (0.7–1.3) ml/mmHg m^2^	
Bobhate, 2015 (21)	CHD-PAH	*N* = 75 4 (0.3–17) years	mPAP: 43 ± 19 mmHg Rp: 9.7 ± 6 Wood units m^2^ Cp: 2.1 ± 0.8 ml/mmHg m^2^	CHD patients with adverse events had Cp < 1.0 ml/mmHg m^2^
Muneuchi, 2016 (13)	VSD/PH	*N* = 100 2.9 (0.6–28.5) months	mPAP: 65 (56–70) mmHg Rp: 2.18 (1.64–3.19) Wood units m^2^ Cp: 2.67 (2.01–3.38) ml/mmHg m^2^	Low preoperative Cp was an independent predictor for higher postoperative pulmonary systolic pressure
Takatsuki, 2017 (22)	Idiopathic/hereditary PAH	*N* = 76 10 ± 3.6 years	mPAP: 68 ± 19 mmHg Rp: 19.9 (7.1–43.0) Wood units m^2^ Cp: 1.1 (0.3–2.0) ml/mmHg m^2^	Low Cp independently correlated with higher BNP level and worse NYHA functional class
Okada, 2017 (23)	Preterm infants/VSD	*N* = 13 77 (13–345) days	mPAP: 53 (47–69) mmHg Rp: 2.3 (1.2–4.8) Wood units m^2^ Cp: 2.1 (1.2–4.5) ml/mmHg m^2^	Preterm infants with VSD/PH had lower Cp than full-term infants, causing a modest increase in pulmonary arterial pressure
Cheng, 2017 (24)	Eisenmenger syndrome	*N* = 48 29 ± 2 years	mPAP: 79 ± 18 mmHg Rp: 18.8 ± 11.2 Wood units m^2^ Cp: 1.4 ± 0.9 ml/mmHg m^2^	CHD-PAH patients with Cp ≤0.84 ml/mmHg m^2^ exhibited worse hemodynamics, including high plasma level of NT-proBNP, elevated mPAP, increased Rp, and low mixed venous oxygen saturation. Cp < 1.04 ml/mmHg m^2^ was independently associated with poor prognosis in adult patients with CHD-PAH, regardless of the clinical phenotypes
	PAH with small defect	*N* = 20 32 ± 11 years	mPAP: 64 ± 20 mmHg Rp: 14.6 ± 9.5 Wood units m^2^ Cp: 1.7 ± 1.1 ml/mmHg m^2^	
	PAH after corrective surgery	*N* = 43 26 ± 9 years	mPAP: 75 ± 27 mmHg Rp: 4.5 ± 2.4 Wood units m^2^ Cp: 1.3 ± 1.1 ml/mmHg m^2^	
Muneuchi, 2019 (18)	CHD-PAH	*N* = 27 4 (2–7) months	mPAP: 53 (36–60) mmHg Rp: 7.47 (3.70–9.59) Wood units m^2^ Cp: 0.99 (0.74–1.42) ml/mmHg m^2^	Cp < 1.22 ml/mmHg m^2^ could independently predict histologically proven pulmonary vascular disease in patients with CHD-PAH
Iwaya, 2019 (25)	Trisomy 21/VSD	*N* = 85 3.0 (1.9–4.0) months	mPAP: 38 (31–45) mmHg Rp: 2.80 (1.76–3.55) Wood units m^2^ Cp: 2.27 (1.62–3.00) ml/mmHg m^2^	Individuals with trisomy 21 had low Cp, which was related to requirement of postoperative home oxygen therapy
Iwaya, 2020 (26)	ASD/PAH	*N* = 22 5 (1–11) months	mPAP: 41 (20–60) mmHg Rp: 4.11 (0.68–15.80) Wood units m^2^ Cp: 1.80 (0.63–6.16) ml/mmHg m^2^	Cp < 0.97 ml/mmHg m^2^ was a predictor of poor outcomes in infants with isolated ASD and severe PAH
Doi, 2021 (27)	AVSD/PH	*N* = 42 65 (47–114) days	mPAP: 36 (29–46) mmHg Rp: 3.45 (2.79–4.98) Wood units m^2^ Cp: 2.78 (1.68–4.10) ml/mmHg m^2^	Cp in infants with complete AVSD were related to gestational age and Down syndrome, although it was not related to the presence of left heart lesions
Hatai, 2022 (28)	Trisomy 18/CHD	*N* = 20 4.6 (3.0–6.9) months	mPAP: 41 (33–49) mmHg Rp: 2.0 (1.6–3.3) Wood units m^2^ Cp: 3.5 (2.3–5.5) ml/mmHg m^2^	Rp and Cp in trisomy 18 subjects significantly differed from those in trisomy 21 subjects and were identical to those observed in subjects without chromosomal anomaly
Iwaya, 2022 (29)	VSD/PH	*N* = 217 2.8 (1.7–4.4) months	mPAP: 36 (28–43) mmHg Rp: 1.95 (1.38–2.59) Wood units m^2^ Cp: 2.98 (2.42–3.88) ml/mmHg m^2^	Pulmonary circulation depended upon Cp rather than Rp according to an increase in pulmonary blood flow despite the constancy in RC time

Continuous values are expressed as the mean ± the standard deviation or the median (the interquartile range).

ASD, atrial septal defect; AVSD, atrioventricular septal defect; BNP, brain natriuretic peptide; CHD, congenital heart disease; Cp, pulmonary vascular capacitance; mPAP, mean pulmonary arterial pressure; NT-proBNP, N-terminal prohormone of brain natriuretic peptide; NYHA, New York Heart Association; PAH, pulmonary arterial hypertension; PH, pulmonary hypertension; RC time, resistor–capacitor time; Rp, pulmonary vascular resistance; VSD, ventricular septal defect; WHO, World Health Organization; 6 MWD, 6-min walk distance.

## Resistor–capacitor coupling in patients with congenital heart disease

**Figure 3:**
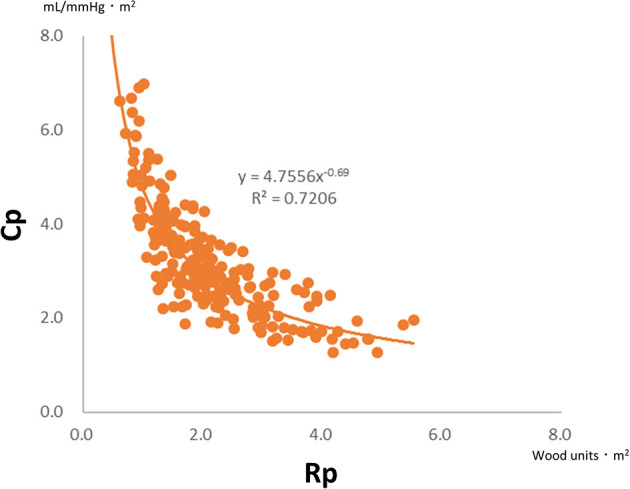
Plots of Rp and Cp in 200 infants with ventricular septal defect and pulmonary hypertension (mean pulmonary arterial pressure >20 mmHg). There is a universal hyperbolic relationship between Rp and Cp. Rp, pulmonary vascular resistance; Cp, pulmonary vascular capacitance.

There is a unique inverse hyperbolic relationship between Rp and Cp when their plots are drawn (Figure 3). As a result, the product of Rp and Cp is constant with units of time, called the time constant or resistor–capacitor (RC) time. The time constant is an exponential pressure decay in diastole, referring to the time until the pulmonary arterial pressure decreases to e^−1^ (37%) of the pulmonary arterial systolic pressure (Figure 1). A higher Rp limits downstream blood flow into the peripheral circulation, while a lower Cp decreases blood flow, which accumulates within the vessels during systole and releases into the peripheral circulation during diastole. In other words, a decay of pulmonary arterial pressure during diastole does not depend on cardiac function but on the condition of the pulmonary vascular bed (8, 32).

**Figure 4:**
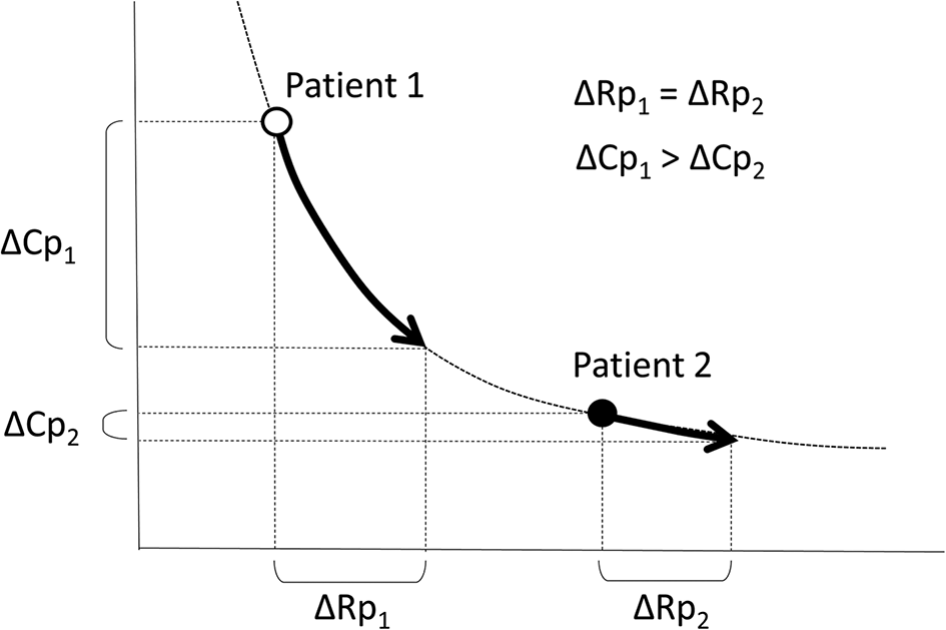
Unique relationship between Rp and Cp. A patient who is developing pulmonary hypertension is placed in the upper left region of the RC coupling curve (Patient 1, open circle) and moves from left to right along the curve. Then, a subtle increase in Rp is accompanied by a substantial decrease in Cp. Meanwhile, in a patent with more advanced stages of the disease (Patient 2, closed circle), Cp has already reached the minimum and any further increase in Rp accompanies no meaningful change in Cp. Rp, pulmonary vascular resistance; Cp, pulmonary vascular capacitance; RC, resistor–capacitor.

**Figure 5:**
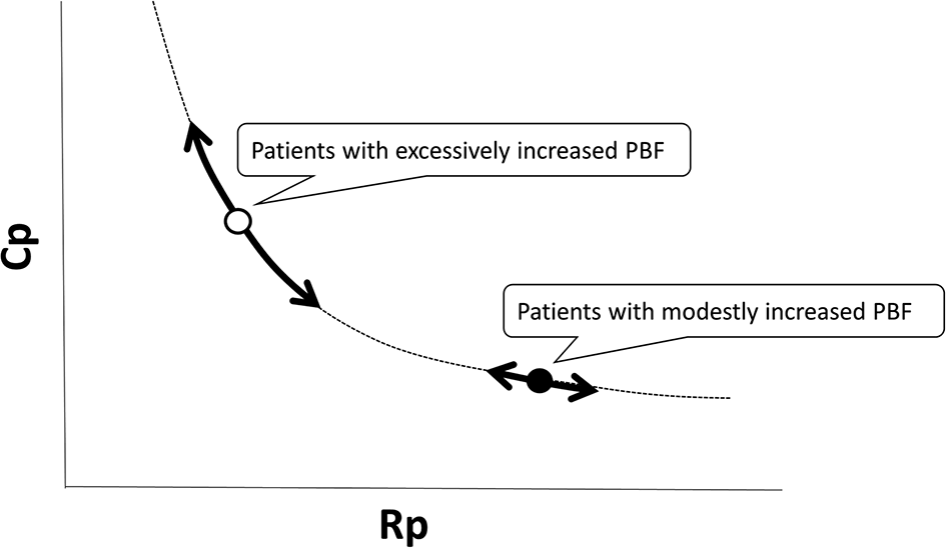
RC coupling among infants with ventricular septal defect and pulmonary hypertension; patients with excessively increased pulmonary blood flow are distributed in the upper left part of the RC coupling curve (open circle), whereas patients with modestly increased pulmonary blood flow are distributed in the lower right part of the curve (closed circle), which suggested that a subtle change in Rp gives a substantial change in Cp in patients with excessive pulmonary blood flow. Rp, pulmonary vascular resistance; Cp, pulmonary vascular capacitance; RC, resistor–capacitor.

The RC coupling curve is helpful in understanding the conditions of the pulmonary vascular bed. Previous reports have shown that RC coupling remains constant after disease-specific therapy in patients with pulmonary arterial hypertension (33). A patient developing pulmonary hypertension is initially placed in the upper left region of the RC coupling curve and moves from left to right along the curve. A subtle increase in Rp is accompanied by a substantial decrease in Cp, which implies that the loss of Cp is an early sign of worsening pulmonary hypertension. In contrast, in more advanced stages of the disease, Cp has already reached a minimum, and any further increase in Rp is accompanied by no meaningful change in Cp (Figure 4) (32, 34). In our previous study on RC coupling among infants with ventricular septal defect, patients with excessively increased pulmonary blood flow were distributed in the upper left part of the RC coupling curve, whereas patients with modestly increased pulmonary blood flow were distributed in the lower right part of the curve, suggesting that a subtle change in Rp gave a substantial change in Cp in patients with excessive pulmonary blood flow (Figure 5) (29). Therefore, decreased Cp is an early sign of reduced effective pulmonary vascular beds before Rp increases in patients with CHD-PH, who exhibit changes in intracardiac shunt amount depending on the pulmonary input impedance. In patients with normal or subtly increased Rp, alterations in Cp become more important than alterations in Rp. Importantly, it is emphasized that the combined assessment of Rp and Cp, which outlines the pulmonary arterial input impedance, is better than each assessment of the two variables separately (35, 36).

**Figure 6:**
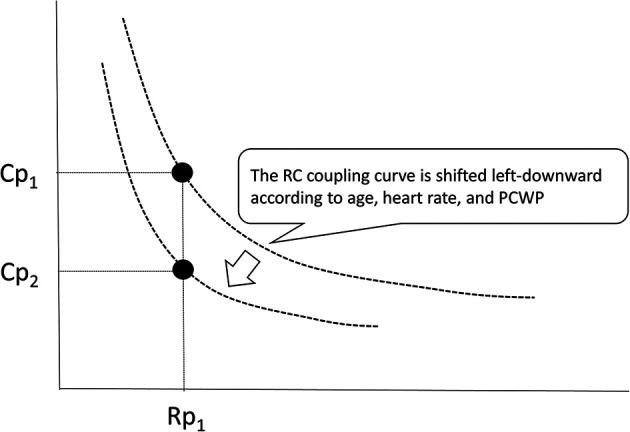
RC coupling curve can be shifted left-downward because the RC time can be altered according to age, heart rate, and pulmonary capillary wedge pressure. When the RC coupling curve is shifted, one value of Rp gives different values of Cp. Rp, pulmonary vascular resistance; Cp, pulmonary vascular capacitance; RC, resistor–capacitor.

Although RC time remains relatively constant across large catheterization cohorts including different patient populations, recent reports have shown that RC time can be altered according to age, heart rate, and pulmonary capillary wedge pressure (Figure 6) (12, 34, 37, 38). Sajan et al. reported that a higher Cp was required to achieve favorable survival in patients with smaller body compositions compared to those with larger ones (14). Therefore, the RC coupling curve can be shifted left-downward among neonates and infants. Age-related differences in RC time are probably due to the properties of the resistance and capacitance vessels according to the body size. There are several reports on heart rate and RC time. Wright et al. reported that exercise was associated with a decrease in Cp and a resulting decline in RC time in healthy individuals (12). Moreira et al. reported that RC time was inversely correlated with heart rate in patients who underwent heart transplantation (39). These findings indicate that an increase in heart rate leads to a decline in RC time. Meanwhile, Reuben et al. reported that disproportional decreases in Cp relative to Rp were observed in patients with mitral valve stenosis, which was due to an increase in smooth muscle tone in the pulmonary arterial walls following elevated left atrial pressure (40). Tedford et al. also found that increasing pulmonary capillary wedge pressure progressively decreased the RC time, effectively enhancing right ventricular pulsatile load in patients with acute and chronic left ventricular filling disturbance (34). These findings suggest that pulmonary capillary wedge pressure acts as the downstream pressure that amplifies peripheral pulse reflections (40). Thus, a higher pulmonary capillary wedge pressure is associated with a low RC time, which means that the RC coupling curve is also shifted left-downward in patients with increased pulmonary capillary wedge pressure (34, 37). On the other hand, we recently reported that RC time remained unchanged in alterations in pulmonary blood flow due to left-to-right shunt in infants with ventricular septal defect (29). Therefore, the RC time in patients with CHD is constant within each patient but varies among patients within a very narrow range because they have different predisposing factors. When an RC coupling curve is shifted left-downward (RC time decreases), the same Rp value will correspond to a lower Cp value (Figure 6). In CHD patients with such a pulmonary vascular condition, hemodynamic modifications, such as the closure of a defect, will result in higher pulmonary arterial pressure than expected. Actually, in infants with ventricular septal defect and pulmonary hypertension, a lower preoperative Cp is associated with elevated postoperative pulmonary arterial pressure even when the preoperative Rp was identical (13, 23, 27), which may allow us to preoperatively identify potential candidates for postoperative targeted therapy for pulmonary hypertension using the combination assessments of Rp and Cp.

So far, only Rp has been believed to be the universal parameter to determine the indication for corrective surgery in patients with CHD-PH. The criterion for operability in the current guideline is based on experts' opinion, which offers that patients with Rp of 4–8 Wood unit m^2^ should be individually evaluated in tertiary centers, while patients with Rp > 8 Wood unit m^2^ are not indicated for corrective surgery and have unfavorable long-term outcomes (41). Acute vasodilator testing during right heart catheterization, using inhaled nitric oxide or intravenous epoprostenol, is recommended to distinguish reversible and progressive changes in the pulmonary vasculatures, although specific criteria for a positive response or specific hemodynamic targets are still lacking. However, the constancy of RC time indicates that a patient's position on an RC coupling curve can predict a therapeutic target (34). When one encounters patients with CHD, such as atrial septal defect or ventricular septal defect, with modestly increased Rp, the assessment of Cp is crucial to determine the treatment strategy. An appropriate Cp value against a given Rp will guarantee satisfied pulmonary hemodynamics after corrective surgery, whereas a lower Cp value against a given Rp will indicate a residue of pulmonary hypertension after corrective surgery, which suggests the possibility that pulmonary vasodilator therapy is required before or after surgery.

Meanwhile, an increase in Cp can be the goal of pulmonary hypertension therapy in patients with CHD-PH. There are several reports on pulmonary vascular targeted therapy for CHD-PH. Previous reports have shown that treatment with an endothelin receptor antagonist, such as bosentan, results in significant improvements in exercise capacity, hemodynamics, and functional class, without negative effects on the overall shunt in patients with Eisenmenger syndrome (42–46). In addition, the use of specific pulmonary vascular target therapies, including bosentan, sildenafil, and epoprostenol, contributes to reduced mortality in patients with Eisenmenger syndrome (47). In these studies, there were no significant changes in pulmonary arterial pressure and Rp before and after treatment; however, no study on targeted therapy in patients with CHD-PH has explored the therapeutic outcome of increased Cp. Thus, a further study targeting the goal of increased Cp is warranted in patients with CHD-PH to clarify the efficacy of specific pulmonary vasodilators.

## Pulmonary vascular resistance and capacitance in the specific populations

Pulmonary circulation in patients with CHD is affected by various factors. First, the RC coupling is altered owing to prematurity. We previously reported that preterm infants with ventricular septal defect and pulmonary hypertension had lower Cp than full-term infants, causing a modest increase in pulmonary arterial pressure (23). Preterm birth is an independent risk factor for developing pulmonary arterial hypertension, and neither birth weight nor small for gestational age is related to pulmonary arterial hypertension (48–50). Low Cp levels in preterm infants may be associated with unusual development and growth of the pulmonary vasculature, including angiogenesis (direct extension of existing vessels) and vasculogenesis (formation of vessels from primitive hemangioblasts). Recent studies have shown that impaired angiogenic signaling results in disrupted vascular growth and abnormal vascular function in preterm infants (51). This arterial dysfunction is hypothesized as “premature aging” theory based on a decrease in capillary recruitment or an increase in extracellular matrix formation during the fetal and neonatal periods, which is attributable to low Cp in this population (23). Therefore, RC coupling should be considered when preterm infants with CHD-PH are treated.

Second, individuals with trisomy 21, who often accompany CHD, have a unique pulmonary vasculature characterized by low Cp compared to subjects without chromosomal anomalies (25, 27). Although individuals with trisomy 21 have an increased risk of developing pulmonary arterial hypertension and require careful management during the perioperative period (52), pulmonary hypertension associated with trisomy 21 is caused by heterogeneous factors, including left-to-right shunt CHD, abnormal pulmonary vasculature growth, hypoxic vasoconstriction due to upper airway obstruction, and an imbalance between pulmonary vasoconstriction and relaxation (53–57). Individuals with trisomy 21 often have micrognathia, midfacial hypoplasia, glossoptosis, abnormal aryepiglottic folds, gastroesophageal reflux, and central hypoventilation, which promote alveolar hypoxic vasoconstriction (52). Furthermore, individuals with trisomy 21 have hypoplasia of the pulmonary vasculature with decreased number of branches and capillaries (52). Histopathological findings of the pulmonary arteries are characterized by poor thickening of the tunica media, which results in high shear stress per unit of the pulmonary arterial media and compensatory intimal thickening, especially with accompanying left-to-right CHD (54, 58). Although mechanisms of abnormal pulmonary vascular development remain uncertain, recent studies have shown that three antiangiogenic genes encoded on chromosome 21, including endostatin, a regulator of calcineurin-1, and β-amyloid peptide, are overexpressed in individuals with trisomy 21, resulting in disruption of angiogenic signals (59, 60). These specific factors lead to a reduction in capillary recruitment, ensuring a left-downward-shifted RC coupling curve. Notably, low preoperative Cp is an independent predictor for the requirement of postoperative home oxygen therapy, which is introduced in 10%–40% patients of patients with CHD and trisomy 21 (25, 27, 61). Meanwhile, in trisomy 18, which is the second most common chromosomal aneuploidy, Rp and Cp values are comparable to those in subjects without chromosomal anomalies (28).

## Future perspective of pulmonary vascular resistance and capacitance

The unique hyperbolic relationship between Rp and Cp demonstrates individual characteristics of the pulmonary vasculature. In this review, we described that alterations in Cp become more prominent than those in Rp in CHD patients with normal values of Rp (<2 Wood units m^2^). We consider that this concept can be applied to different CHDs, including those with decreased pulmonary blood flow, such as tetralogy of Fallot (TOF). Bédard et al. reported that patients with TOF exhibited histological abnormalities of the pulmonary arteries, including medial necrosis, fibrosis, cyst-like formation, and abnormal elastic tissue accumulation (62), which may be responsible for alterations in Cp. Inuzuka et al. reported that patients with repaired TOF demonstrated low Cp, which enhanced wave reflection and high pulsatile right ventricular workload. In addition, they addressed that low Cp was a significant predictor of right ventricular dilatation due to pulmonary regurgitation and stenosis (63). In patients with pulmonary atresia, ventricular septal defect, and major aortopulmonary collateral arteries (PAVSD/MAPCA), the pulmonary circulation is more confounded by abnormal pulmonary arborization following segmental pulmonary hypertension (64). Grosse-Wortmann et al. reported that preoperative total pulmonary blood flow was inversely correlated with postoperative right ventricular systolic pressure in patients with PAVSD/MAPCA (65). These findings suggest the possibility that total Cp can represent the pulmonary input impedance in CHD patients with different pulmonary vascular conditions, although the precise Rp cannot be determined in each arborized area. Therefore, assessment of both preoperative Rp and Cp may be useful for predicting postoperative pulmonary input impedance in patients with different categories of CHD.

Although the relationship between Rp and Cp represented by the Windkessel model conceptualizes the sums of steady and pulsatile afterload against right ventricular work in pulsatile circulation, it may be applied to evaluate pulmonary circulation even in CHD patients with nonpulsatile pulmonary blood, such as patients who undergo the Glenn or Fontan procedure. Simulation models represented by the electrical circuit analog show that Cp is an important parameter (66, 67). Senzaki et al. reported that Cp, estimated by pulmonary arterial size, affected postoperative hemodynamics, including elevated central venous pressure and total pulmonary impedance, in patients who underwent the Fontan procedure. Furthermore, the pulmonary input impedance abruptly increased when the pulmonary arterial index determined by the right and left pulmonary arterial diameters was less than 100 mm^2^/m^2^ (66). These findings suggest that Cp also plays an important role in the nonpulsatile pulmonary circulation.

Although patients after the Glenn or Fontan procedure do not usually fulfill the definition of pulmonary hypertension with mean pulmonary arterial pressure >20 mmHg, they occasionally have variable degrees of pulmonary vascular disease (68, 69). However, definitive criteria for pulmonary hypertension have not been established among patients who undergo the Glenn or Fontan procedure. It is assumed that the combined assessment of Rp and Cp may be helpful in detecting pulmonary vascular disease. Previous studies regarding pulmonary vasodilator therapy in patients undergoing the Fontan procedure had targeted improvements in clinical symptoms and exercise tolerance (70, 71), although hemodynamic parameters, including pulmonary arterial pressure and Rp, remained unchanged after interventions. However, it is possible that a prospective study that targets an increase in Cp will provide novel evidence to determine the effectiveness of pulmonary vasodilators, and Cp is expected to be a potential target for treatment in patients with CHD-PH.

## Conclusions

The pulmonary artery input impedance consists of steady and pulsatile components representing Rp and Cp, respectively. Alterations in Cp more strictly reflect conditions of the pulmonary vasculature than those in Rp in CHD patients at an early stage of developing pulmonary hypertension or with excessive pulmonary blood flow due to a left-to-right shunt. Therefore, comprehensive assessments of the pulmonary circulation using the combination of Rp and Cp are necessary in patients with CHD-PH who have heterogeneous categories of pulmonary hypertension. In addition, Cp is expected to be a potential target for treating patients with CHD-PH.

## References

[1] Astrand PO Cuddy TE Saltin B Stenberg J. Cardiac output during submaximal and maximal work. J Appl Physiol. (1964) 19:268–74. 10.1152/jappl.1964.19.2.268 14155294

[2] Kovacs G Berghold A Scheidl S Olschewski H. Pulmonary arterial pressure during rest and exercise in healthy subjects: a systematic review. Eur Respir J. (2009) 34:888–94. 10.1183/09031936.00145608 19324955

[3] Beshay S Sahay S Humbert M. Evaluation and management of pulmonary arterial hypertension. Respir Med. (2020) 171:106099. 10.1016/j.rmed.2020.106099 32829182

[4] Galiè N Hoeper MM Humbert M Torbicki A Vachiery JL Barbera JA Guidelines for the diagnosis and treatment of pulmonary hypertension: the Task Force for the Diagnosis and Treatment of Pulmonary Hypertension of the European Society of Cardiology (ESC) and the European Respiratory Society (ERS), endorsed by the International Society of Heart and Lung Transplantation (ISHLT). Eur Heart J. (2009) 30:2493–537. 10.1093/eurheartj/ehp297 19713419

[5] Frank O. The basic shape of the arterial pulse. First treatise: mathematical analysis. 1899. J Mol Cell Cardiol. (1990) 22:255–77. 10.1016/0022-2828(90)91460-o 21438422

[6] Sanz J Kariisa M Dellegrottaglie S Prat-González S Garcia MJ Fuster V Evaluation of pulmonary artery stiffness in pulmonary hypertension with cardiac magnetic resonance. JACC Cardiovasc Imaging. (2009) 2:286–95. 10.1016/j.jcmg.2008.08.007 19356573

[7] Linehan JH Dawson CA Rickaby DA Bronikowski TA. Pulmonary vascular compliance and viscoelasticity. J Appl Physiol (1985). (1986) 61:1802–14. 10.1152/jappl.1986.61.5.1802 3781989

[8] Saouti N Westerhof N Postmus PE Vonk-Noordegraaf A. The arterial load in pulmonary hypertension. Eur Respir Rev. (2010) 19:197–203. 10.1183/09059180.00002210 20956192 PMC9487275

[9] Segers P Brimioulle S Stergiopulos N Westerhof N Naeije R Maggiorini M Pulmonary arterial compliance in dogs and pigs: the three-element windkessel model revisited. Am J Physiol. (1999) 277:H725–31. 10.1152/ajpheart.1999.277.2.H725 10444499

[10] Stergiopulos N Segers P Westerhof N. Use of pulse pressure method for estimating total arterial compliance in vivo. Am J Physiol. (1999) 276:H424–8. 10.1152/ajpheart.1999.276.2.H424 9950841

[11] Vulliémoz S Stergiopulos N Meuli R. Estimation of local aortic elastic properties with MRI. Magn Reson Med. (2002) 47:649–54. 10.1002/mrm.10100 11948725

[12] Wright SP Granton JT Esfandiari S Goodman JM Mak S. The relationship of pulmonary vascular resistance and compliance to pulmonary artery wedge pressure during submaximal exercise in healthy older adults. J Physiol. (2016) 594:3307–15. 10.1113/JP271788 26880530 PMC4824842

[13] Muneuchi J Nagatomo Y Watanabe M Joo K Onzuka T Ochiai Y Relationship between pulmonary arterial resistance and compliance among patients with pulmonary arterial hypertension and congenital heart disease. J Thorac Cardiovasc Surg. (2016) 152:507–13. 10.1016/j.jtcvs.2016.03.080 27189891

[14] Sajan I Manlhiot C Reyes J McCrindle BW Humpl T Friedberg MK. Pulmonary arterial capacitance in children with idiopathic pulmonary arterial hypertension and pulmonary arterial hypertension associated with congenital heart disease: relation to pulmonary vascular resistance, exercise capacity, and survival. Am Heart J. (2011) 162:562–8. 10.1016/j.ahj.2011.06.014 21884877

[15] Saouti N Westerhof N Helderman F Marcus JT Stergiopulos N Westerhof BE RC Time constant of single lung equals that of both lungs together: a study in chronic thromboembolic pulmonary hypertension. Am J Physiol Heart Circ Physiol. (2009) 297:H2154–60. 10.1152/ajpheart.00694.2009 19801491

[16] Presson RG Jr Baumgartner WA Jr Peterson AJ Glenny RW Wagner WW Jr. Pulmonary capillaries are recruited during pulsatile flow. J Appl Physiol (1985). (2002) 92:1183–90. 10.1152/japplphysiol.00845.2001 11842057

[17] Langleben D Fox BD Orfanos SE Giovinazzo M Catravas JD. Pulmonary capillary recruitment and distention in mammalian lungs: species similarities. Eur Respir Rev. (2022) 31:210248. 10.1183/16000617.0248-2021 35197268 PMC9489178

[18] Muneuchi J Ochiai Y Masaki N Okada S Iida C Sugitani Y Pulmonary arterial compliance is a useful predictor of pulmonary vascular disease in congenital heart disease. Heart Vessels. (2019) 34:470–6. 10.1007/s00380-018-1263-9 30225809

[19] Basnet NB Awa S Hishi T Yanagisawa M. Pulmonary arterial compliance in children with atrial and ventricular septal defect. Heart Vessels. (2000) 15:61–9. 10.1007/s003800070033 11199505

[20] Douwes JM Roofthooft MT Bartelds B Talsma MD Hillege HL Berger RM. Pulsatile haemodynamic parameters are predictors of survival in paediatric pulmonary arterial hypertension. Int J Cardiol. (2013) 168:1370–7. 10.1016/j.ijcard.2012.12.080 23340486

[21] Bobhate P Guo L Jain S Haugen R Coe JY Cave D Cardiac catheterization in children with pulmonary hypertensive vascular disease. Pediatr Cardiol. (2015) 36:873–9. 10.1007/s00246-015-1100-1 25577228

[22] Takatsuki S Nakayama T Ikehara S Matsuura H Ivy DD Saji T. Pulmonary arterial capacitance index is a strong predictor for adverse outcome in children with idiopathic and heritable pulmonary arterial hypertension. J Pediatr. (2017) 180:75–9.e2. 10.1016/j.jpeds.2016.10.003 27810156

[23] Okada S Muneuchi J Nagatomo Y Watanabe M Iida C Shirouzu H Pulmonary arterial resistance and compliance in preterm infants. Int J Cardiol. (2017) 244:265–70. 10.1016/j.ijcard.2017.06.056 28637627

[24] Cheng XL Liu ZH Gu Q Ni XH Luo Q Zhao ZH Prognostic value of pulmonary artery compliance in patients with pulmonary arterial hypertension associated with adult congenital heart disease. Int Heart J. (2017) 58:731–8. 10.1536/ihj.16-449 28966315

[25] Iwaya Y Muneuchi J Inoue Y Watanabe M Okada S Ochiai Y. Relationship between pulmonary arterial resistance and compliance in patients with down syndrome. Pediatr Cardiol. (2019) 40:841–7. 10.1007/s00246-019-02080-9 30830280

[26] Iwaya Y Muneuchi J Watanabe M Sugitani Y Ochiai Y. Decreased pulmonary arterial compliance is a predictor for poor outcomes in infants with isolated atrial septal defect and pulmonary hypertension. Pediatr Cardiol. (2020) 41:1408–13. 10.1007/s00246-020-02400-4 32556489

[27] Doi H Muneuchi J Watanabe M Sugitani Y Matsuoka R Ezaki H Characteristics of the pulmonary circulation in infants with complete atrioventricular septal defect. Cardiol Young. (2021) 31:556–61. 10.1017/S1047951120004424 33303047

[28] Hatai E Muneuchi J Sugitani Y Doi H Furuta T Ezaki H Pulmonary vascular resistance and compliance in individuals with trisomy 18. Am J Med Genet A. (2022) 188:534–9. 10.1002/ajmg.a.62550 34729911

[29] Iwaya Y Muneuchi J Sugitani Y Watanabe M. Pulmonary vascular resistance and compliance in pulmonary blood flow alterations in children with congenital heart disease. Heart Vessels. (2022) 37:1283–9. 10.1007/s00380-021-02009-4 35001144

[30] Mahapatra S Nishimura RA Oh JK McGoon MD. The prognostic value of pulmonary vascular capacitance determined by Doppler echocardiography in patients with pulmonary arterial hypertension. J Am Soc Echocardiogr. (2006) 19:1045–50. 10.1016/j.echo.2006.03.008 16880101

[31] Mahapatra S Nishimura RA Sorajja P Cha S McGoon MD. Relationship of pulmonary arterial capacitance and mortality in idiopathic pulmonary arterial hypertension. J Am Coll Cardiol. (2006) 47:799–803. 10.1016/j.jacc.2005.09.054 16487848

[32] Ghio S Schirinzi S Pica S. Pulmonary arterial compliance: how and why should we measure it? Glob Cardiol Sci Pract. (2015) 2015:58. 10.5339/gcsp.2015.58 26779530 PMC4710864

[33] Lankhaar JW Westerhof N Faes TJ Gan CT Marques KM Boonstra A Pulmonary vascular resistance and compliance stay inversely related during treatment of pulmonary hypertension. Eur Heart J. (2008) 29:1688–95. 10.1093/eurheartj/ehn103 18349027

[34] Tedford RJ Hassoun PM Mathai SC Girgis RE Russell SD Thiemann DR Pulmonary capillary wedge pressure augments right ventricular pulsatile loading. Circulation. (2012) 125:289–97. 10.1161/CIRCULATIONAHA.111.051540 22131357 PMC3264431

[35] Wang Z Chesler NC. Pulmonary vascular wall stiffness: an important contributor to the increased right ventricular afterload with pulmonary hypertension. Pulm Circ. (2011) 1:212–23. 10.4103/2045-8932.83453 22034607 PMC3198648

[36] Lankhaar JW Westerhof N Faes TJ Marques KM Marcus JT Postmus PE Quantification of right ventricular afterload in patients with and without pulmonary hypertension. Am J Physiol Heart Circ Physiol. (2006) 291:H1731–7. 10.1152/ajpheart.00336.2006 16699074

[37] Najjar E Lund LH Hage C Nagy AI Johnson J Manouras A. The differential impact of the left atrial pressure components on pulmonary arterial compliance-resistance relationship in heart failure. J Card Fail. (2021) 27:277–85. 10.1016/j.cardfail.2020.09.008 32956814

[38] Hadinnapola C Li Q Su L Pepke-Zaba J Toshner M. The resistance-compliance product of the pulmonary circulation varies in health and pulmonary vascular disease. Physiol Rep. (2015) 3:e12363. 10.14814/phy2.12363 25902784 PMC4425968

[39] Moreira N Baptista R Costa S Franco F Pêgo M Antunes M. Lowering pulmonary wedge pressure after heart transplant: pulmonary compliance and resistance effect. Arq Bras Cardiol. (2015) 105:292–300. 10.5935/abc.20150083 26247246 PMC4592178

[40] Reuben SR. Compliance of the human pulmonary arterial system in disease. Circ Res. (1971) 29:40–50. 10.1161/01.res.29.1.40 5561407

[41] Rosenzweig EB Abman SH Adatia I Beghetti M Bonnet D Haworth S Paediatric pulmonary arterial hypertension: updates on definition, classification, diagnostics and management. Eur Respir J. (2019) 53:1801916. 10.1183/13993003.01916-2018 30545978 PMC6351335

[42] Diller GP Dimopoulos K Kaya MG Harries C Uebing A Li W Long-term safety, tolerability and efficacy of bosentan in adults with pulmonary arterial hypertension associated with congenital heart disease. Heart. (2007) 93:974–6. 10.1136/hrt.2006.089185 17639112 PMC1994431

[43] Monfredi O Griffiths L Clarke B Mahadevan VS. Efficacy and safety of bosentan for pulmonary arterial hypertension in adults with congenital heart disease. Am J Cardiol. (2011) 108:1483–8. 10.1016/j.amjcard.2011.07.006 21943933

[44] D’Alto M Romeo E Argiento P D’Andrea A Sarubbi B Correra A Therapy for pulmonary arterial hypertension due to congenital heart disease and Down’s syndrome. Int J Cardiol. (2013) 164:323–6. 10.1016/j.ijcard.2011.07.009 21802156

[45] Vis JC Duffels MG Mulder P de Bruin-Bon RH Bouma BJ Berger RM Prolonged beneficial effect of bosentan treatment and 4-year survival rates in adult patients with pulmonary arterial hypertension associated with congenital heart disease. Int J Cardiol. (2013) 164:64–9. 10.1016/j.ijcard.2011.06.064 21723630

[46] Kaya MG Lam YY Erer B Ayhan S Vatankulu MA Nurkalem Z Long-term effect of bosentan therapy on cardiac function and symptomatic benefits in adult patients with Eisenmenger syndrome. J Card Fail. (2012) 18:379–84. 10.1016/j.cardfail.2012.02.004 22555267

[47] Dimopoulos K Inuzuka R Goletto S Giannakoulas G Swan L Wort SJ Improved survival among patients with Eisenmenger syndrome receiving advanced therapy for pulmonary arterial hypertension. Circulation. (2010) 121:20–5. 10.1161/CIRCULATIONAHA.109.883876 20026774

[48] Naumburg E Söderström L Huber D Axelsson I. Risk factors for pulmonary arterial hypertension in children and young adults. Pediatr Pulmonol. (2017) 52:636–41. 10.1002/ppul.23633 27801982

[49] Dodson RB Rozance PJ Petrash CC Hunter KS Ferguson VL. Thoracic and abdominal aortas stiffen through unique extracellular matrix changes in intrauterine growth restricted fetal sheep. Am J Physiol Heart Circ Physiol. (2014) 306:H429–37. 10.1152/ajpheart.00472.2013 24322609 PMC3920138

[50] Nilsson PM Lurbe E Laurent S. The early life origins of vascular ageing and cardiovascular risk: the EVA syndrome. J Hypertens. (2008) 26:1049–57. 10.1097/HJH.0b013e3282f82c3e 18475139

[51] Baker CD Abman SH Mourani PM. Pulmonary hypertension in preterm infants with bronchopulmonary dysplasia. Pediatr Allergy Immunol Pulmonol. (2014) 27:8–16. 10.1089/ped.2013.0323 24669351 PMC3961769

[52] Saji T. Clinical characteristics of pulmonary arterial hypertension associated with Down syndrome. Pediatr Int. (2014) 56:297–303. 10.1111/ped.12349 24689825

[53] Cooney TP Thurlbeck WM. Pulmonary hypoplasia in Down’s syndrome. N Engl J Med. (1982) 307:1170–3. 10.1056/NEJM198211043071902 6214715

[54] Yamaki S Horiuchi T Sekino Y. Quantitative analysis of pulmonary vascular disease in simple cardiac anomalies with the Down syndrome. Am J Cardiol. (1983) 51:1502–6. 10.1016/0002-9149(83)90665-3 6221650

[55] de Miguel-Díez J Villa-Asensi JR Alvarez-Sala JL. Prevalence of sleep-disordered breathing in children with Down syndrome: polygraphic findings in 108 children. Sleep. (2003) 26:1006–9. 10.1093/sleep/26.8.1006 14746382

[56] Cua CL Rogers LK Chicoine LG Augustine M Jin Y Nash PL Down syndrome patients with pulmonary hypertension have elevated plasma levels of asymmetric dimethylarginine. Eur J Pediatr. (2011) 170:859–63. 10.1007/s00431-010-1361-x 21120524

[57] Fukushima H Kosaki K Sato R Yagihashi T Gatayama R Kodo K Mechanisms underlying early development of pulmonary vascular obstructive disease in Down syndrome: an imbalance in biosynthesis of thromboxane A2 and prostacyclin. Am J Med Genet A. (2010) 152A:1919–24. 10.1002/ajmg.a.33555 20583254

[58] Suzuki K Yamaki S Mimori S Murakami Y Mori K Takahashi Y Pulmonary vascular disease in Down’s syndrome with complete atrioventricular septal defect. Am J Cardiol. (2000) 86:434–7. 10.1016/s0002-9149(00)00960-7 10946038

[59] Sánchez O Domínguez C Ruiz A Ribera I Alijotas J Cabero L Angiogenic gene expression in down syndrome fetal hearts. Fetal Diagn Ther. (2016) 40:21–7. 10.1159/000441356 26513650

[60] Galambos C Minic AD Bush D Nguyen D Dodson B Seedorf G Increased lung expression of anti-angiogenic factors in down syndrome: potential role in abnormal lung vascular growth and the risk for pulmonary hypertension. PLoS One. (2016) 11:e0159005. 10.1371/journal.pone.0159005 27487163 PMC4972384

[61] Hoashi T Hirahara N Murakami A Hirata Y Ichikawa H Kobayashi J Current surgical outcomes of congenital heart surgery for patients with down syndrome in Japan. Circ J. (2018) 82:403–8. 10.1253/circj.CJ-17-0483 28904256

[62] Bédard E McCarthy KP Dimopoulos K Giannakoulas G Gatzoulis MA Ho SY. Structural abnormalities of the pulmonary trunk in tetralogy of Fallot and potential clinical implications: a morphological study. J Am Coll Cardiol. (2009) 54:1883–90. 10.1016/j.jacc.2009.06.040 19892240

[63] Inuzuka R Seki M Sugimoto M Saiki H Masutani S Senzaki H. Pulmonary arterial wall stiffness and its impact on right ventricular afterload in patients with repaired tetralogy of Fallot. Ann Thorac Surg. (2013) 96(4):1435–41. 10.1016/j.athoracsur.2013.05.085 23972390

[64] Yasuhara J Yamagishi H. Pulmonary arterial hypertension associated with tetralogy of Fallot. Int Heart J. (2015) 56(Suppl):S17–21. 10.1536/ihj.14-351 25787793

[65] Grosse-Wortmann L Yoo SJ van Arsdell G Chetan D Macdonald C Benson L Preoperative total pulmonary blood flow predicts right ventricular pressure in patients early after complete repair of tetralogy of Fallot and pulmonary atresia with major aortopulmonary collateral arteries. J Thorac Cardiovasc Surg. (2013) 146:1185–90. 10.1016/j.jtcvs.2013.01.032 23414777

[66] Senzaki H Isoda T Ishizawa A Hishi T. Reconsideration of criteria for the Fontan operation. Influence of pulmonary artery size on postoperative hemodynamics of the Fontan operation. Circulation. (1994) 89:266–71. 10.1161/01.cir.89.1.266 8281656

[67] Liang F Senzaki H Kurishima C Sughimoto K Inuzuka R Liu H. Hemodynamic performance of the Fontan circulation compared with a normal biventricular circulation: a computational model study. Am J Physiol Heart Circ Physiol. (2014) 307:H1056–72. 10.1152/ajpheart.00245.2014 25063796

[68] Ridderbos FJ Wolff D Timmer A van Melle JP Ebels T Dickinson MG Adverse pulmonary vascular remodeling in the Fontan circulation. J Heart Lung Transplant. (2015) 34:404–13. 10.1016/j.healun.2015.01.005 25813767

[69] Lévy M Danel C Tamisier D Vouhé P Leca F. Histomorphometric analysis of pulmonary vessels in single ventricle for better selection of patients for the Fontan operation. J Thorac Cardiovasc Surg. (2002) 123:263–70. 10.1067/mtc.2002.119697 11828285

[70] Li D Zhou X An Q Feng Y. Pulmonary vasodilator therapy after the Fontan procedure: a meta-analysis. Heart Fail Rev. (2021) 26:91–100. 10.1007/s10741-019-09905-y 31845048

[71] Becker K Uebing A Hansen JH. Pulmonary vascular disease in Fontan circulation-is there a rationale for pulmonary vasodilator therapies? Cardiovasc Diagn Ther. (2021) 11:1111–21. 10.21037/cdt-20-431 34527537 PMC8410499

